# Metalloproteins and apolipoprotein C: candidate plasma biomarkers of T2DM screened by comparative proteomics and lipidomics in ZDF rats

**DOI:** 10.1186/s12986-020-00488-2

**Published:** 2020-08-12

**Authors:** Shuai Wang, Zhiyuan Lu, Yuxin Wang, Tianran Zhang, Xiaodong He

**Affiliations:** 1 Institute of Toxicology, School of Public Health, Cheeloo College of Medicine, Shandong University, Jinan, 250012 Shandong China; 2 Key Laboratory of Chemical Biology (Ministry of Education), School of Pharmaceutical Sciences, Cheeloo College of Medicine, Shandong University, Jinan, 250012 Shandong China; 3 Department of Physical and Chemical Inspection, School of Public Health, Cheeloo College of Medicine, Shandong University, Jinan, 250012 Shandong China; 4 Shandong Provincial Key Laboratory of Infection and Immunity, School of Basic Medical Sciences, Cheeloo College of Medicine, Shandong University, 44 West Wenhua Road, Jinan, 250012 Shandong China

**Keywords:** Type 2 diabetes mellitus, Zucker diabetic fatty rats, Proteomics, Lipidomics, Biomarkers

## Abstract

**Background:**

Early diagnosis of type 2 diabetes mellitus (T2DM) is still difficult. Screening of plasma biomarkers has great significance of optimizing diagnosis and predicting the complications of T2DM.

**Methods:**

We used a special diet, Purina #5008, to induce diabetes in Zucker leptin receptor gene-deficient rats (fa/fa) to establish Zucker diabetic fatty (ZDF) rats, simulating the early stage of T2DM. The differentially expressed proteins (DEP) and lipids (DEL), as potential biomarkers, were screened to compare the plasma expression levels in ZDF rats and their basic diet-fed wild-type controls (fa/+) by Tandem Mass Tags (TMT) and liquid chromatography-tandem mass spectrometry.

**Results:**

These two groups had different plasma proteins and lipids profiles consisting of 84 DEPs and, 179 DELs identified in the positive ion mode and 178 DELs in the negative ion mode, respectively. Enrichment analysis of these different indicators showed that oxidative stress, insulin resistance and metabolic disorders of glycan and lipid played an important role in generating the difference. Some markers can be used as candidate biomarkers in prediction and treatments of T2DM, such as ceruloplasmin, apolipoprotein C-I, apolipoprotein C-II and apolipoprotein C-IV.

**Conclusion:**

These plasma differences help to optimize the diagnosis and predict the complications of T2DM, although this remains to be verified in the crowd. Trace elements related-metalloproteins, such as ceruloplasmin, and lipid metabolism and transport-related apolipoprotein C are expected to be candidate biomarkers of T2DM and should be given more attention.

## Background

Diabetes, one of the leading causes of death in the world, is a chronic, metabolic disease characterized by elevated levels of blood glucose. Based on the investigation from the International Diabetes Federation (IDF) Atlas, about 425 million adults worldwide aged 20–79 years are affected by diabetes in 2017 [1]. Most of the diabetics (90–95%) suffering from type 2 diabetes mellitus (T2DM) could benefit directly from early diagnosis and treatments [2]. However, unfortunately, half of the patients may be undiagnosed due to the lack of early detection [1]. The conventional methods based on blood glucose testing need to be improved or supplemented with other diagnostic methods. Moreover, with the obese population and the prevalence of T2DM growing rapidly [3–6], the necessity for prompt diagnosis or prediction of T2DM becomes more urgent.

The development of diseases is accompanied by metabolic changes, and existing studies have shown that biomarkers in plasma and urine can predict the occurrence of some chronic diseases [7–9]. This helps to optimize the diagnostic method and to predict related complications. The biomarkers research regarding diabetes nephropathy, a serious complication of diabetes, has made great progress [10, 11], however, study of early diagnosis of T2DM is still limited [12]. To a certain extent, this is due to that the presence of severe metabolic disorders and signs of microvascular damage in the stage of diabetic complications help in the selection of markers; while slight changes in blood glucose and other metabolites in the early stages of diabetes are not likely to be discovered by epidemiological studies. Therefore, screen potential biomarkers in diabetes animal models is an indispensable step for improving early diagnosis of T2DM.

Zucker diabetic fatty (ZDF) rats are commonly used as spontaneous T2DM animal models and are highly recognized in the development of diabetes drugs [13–16]. Due to the defection of leptin receptor-gene, they show characteristics such as obesity, hyperglycemia, insulin disorders and dyslipidemia in the case of special diet induction, which closely match the pathological characteristics of T2DM patients. This diet-only modeling method is similar to natural development of T2DM in human and does not change the physiological state of rats which may change in experimental diabetes animal models due to drug or surgery. This is of great significance to the screening of candidate biomarkers of T2DM and provides feasibility for our study. The liquid chromatography-tandem mass spectrometry (LC-MS/MS) technology also provides a reliable mean for plasma proteomics and lipidomics. In preliminaries screening of plasma differentially expressed proteins (DEP) and lipids (DEL) in ZDF rats, this study provides an important reference for screening and verification of T2DM plasma biomarkers in the crowd.

## Methods

### Animals and groups

Zucker leptin receptor gene-deficient rats (fa/fa) and their littermate wild-type rats (fa/+) (male, 8 weeks of age, SPF VAF/Elite) were supplied by Charles River in Beijing, China. All animals were kept in a barrier system. The animal room was maintained at approximately 22 °C and 50% humidity with a 12 h light/dark cycle. Food and drinking water were available. Purina #5008 (protein 23.5%, fat (ether extract) 6.5%, fat (acid hydrolysis) 7.5%, fiber (crude) 3.8%, nitrogen-free extract (by difference) 49.4%, ash 6.8%; gross energy 4.15 kcal/gm. Calories provided by the calorigenic nutrients: protein 26.849%, fat (ether extract) 16.710%, carbohydrates 56.441%.) was utilized to induce obesity and diabetes in Zucker leptin receptor gene-deficient rats (fa/fa). Simply, they were fed by Purina #5008, starting at 8 weeks of age, for 3 weeks. Blood glucose > 11.1 mmol/L was used as the standard of successfully modeling of ZDF rats [17]. In order to avoid the diet effects on plasma, the ZDF rats were maintained on a basic diet (crude protein ≥18%, crude fat ≥4%; gross energy 3.40 kcal/gm. Calories provided by the calorigenic nutrients: protein 23.07%, fat (ether extract) 11.85%, carbohydrates 65.08%.) for 1 week, that was the 12th weeks. The wild-type rats (fa/+) were kept on a basic diet all along. By the end of the 12th week, all animals were fasted for 12 h, anesthetized and blooded from the abdominal aorta using EDTA-K_2_ anticoagulation tubes. Plasma was collected after standing and centrifugation, and then stored at − 80 °C until detection. Three samples from each of the ZDF group and their basic diet-fed littermate wild-type group were labeled with TMT to analyze the proteins in plasma by LC-MS/MS. And six from each were used to analyze the lipids by LC-MS/MS. All animals were treated according to the NIH Guide for Care and Use of Laboratory Animals. All protocols were approved by the Institutional Animal Care and Use Committee of Shandong University.

### Proteomic TMT labeling and LC-MS/MS analysis

Proteomic TMT labeling technology used isotopically labeled peptides to analyze the protein levels in groups by high-precision mass spectrometer [18]. The experimental procedures in our study included: extraction, quantification, detection, removal of peak protein, enzyme digestion and desalting [19], labeling, fraction separation and mass spectrometry [20], etc. Reagents and procedures were described in the Additional file 1.

### Proteins identification and screening of differentially expressed proteins (DEP)

The mass data was directly imported into Proteome Discoverer 2.2 for database search. The database we used was the Uniport (Accessed 18 January 2019, *Rattus norvegicus*, 36,090 sequences). Analysis parameters were described carefully in the Additional file 1. Peptides with a confidence of more than 95% were peptides spectrum matches (PSMs). Proteins containing at least one unique peptide were trusted proteins. We screened the results and retained only the PSMs and trusted proteins. FDR validation was also performed to remove peptides and proteins with *P*-value above 5%. Relative protein quantification was performed based on the peak area. The ratio of the mean quantization of the ZDF group to their basic diet-fed littermate wild-type group was the fold change (FC). We considered FC > 1.2 and *P* < 0.05 as DEPs.

### DEPs enrichment analysis

Gene Ontology (GO), Cluster of Orthologous Groups of proteins (COG), Kyoto Encyclopedia of Genes and Genomes (KEGG) annotations, and Inter Pro (IPR) annotations [21–25] were performed to fully understand the functional properties of DEPs.

GO function enrichment analysis was carried out to identify the functional process of the DEPs in biological processes (BP), cell composition (CC) and molecular function (MF) by hypergeometric verification. KEGG pathway enrichment analysis was also conducted for exploring the causes of DEPs and the mechanisms of T2DM. *P* < 0.05 was identified as the significant difference.

### Protein-protein interaction (PPI) network analysis of DEPs

PPI network analysis of the DEPs was constructed from the STRING (https://string-db.org) and visualized by Cytoscape (version 3.7.1). The Molecular Complex Detection (MCODE, version 1.31) app in Cytoscape was used to analyze the modules in the network.

### Lipidomic LC-MS/MS analysis

The LC-MS/MS technique was used for lipidomics research. The experimental procedures included: lipid extraction, LC-MS/MS detection [26–30], etc. The reagents and procedures were detailed in the Additional file 1.

### Lipids identification and screening of differentially expressed lipids (DEL)

Progenesis QI (Waters) was used to identify lipids and multivariate statistical analysis. Lipidmaps (http://www.lipidmaps.org), HMDB (http://www.hmdb.ca), NIST (https://chemdata.nist.gov) and an in-house lipid database of Novogene Bioinformatics Technology Co. Ltd. were used for identification. Reagents and procedures are also described in the Additional file 1. The multivariate statistical analyses used to reveal the differences included principal component analysis (PCA) and partial least squares discriminant analysis (PLS-DA). The variable importance in the projection (VIP) of the first principal component of the PLS-DA model was combined with *P* of T-test to screen DELs. We considered VIP > 1.0, FC > 2.0 and *P* < 0.05 as DELs.

### Correlation analysis of proteomics and lipidomics

According to the order of FC, we selected the top 50 DEPs and the top 20 DELs for statistical correlation analysis of expression levels to explore the consistency of the proteomic and lipidomic data. We also conducted KEGG pathway enrichment analysis on DELs, reviewed and compared the results of DEPs and DELs. The pathways in which both proteins and lipids were enriched had received particular attention.

## Results

### Purina #5008 diet-induced irreversible diabetes in Zucker leptin receptor gene-deficient rats

**Fig. 1:**
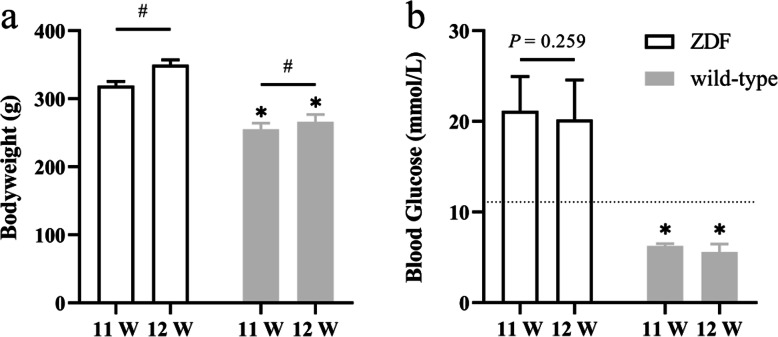
Purina #5008 diet-induced irreversible diabetes in Zucker leptin receptor gene-deficient rats. **a** Bodyweight of the rats. **b** Blood glucose of the rats. The dotted line in the figure represents the standard for successful modeling of ZDF, the blood glucose > 11.1 mmol/L. (*n* = 10. #: *P* < 0.05, paired T-test in 11 W and 12 W. *: *P* < 0.05, paired T-test in ZDF and their basic diet-fed littermate wild-type control at the same time point)

After fed by Purina #5008 for 3 weeks, up to 11 weeks old, Zucker leptin receptor gene-deficient rats (fa/fa) developed obesity and elevated blood glucose (Fig. 1). And this early diabetic state was not corrected by 1 week’s basic diet, that was when they were 12 weeks old (*n* = 10, paired T-test in 11 W and 12 W, *P* = 0.259).

### Screening of DEPs and their enrichment analysis

**Fig. 2:**
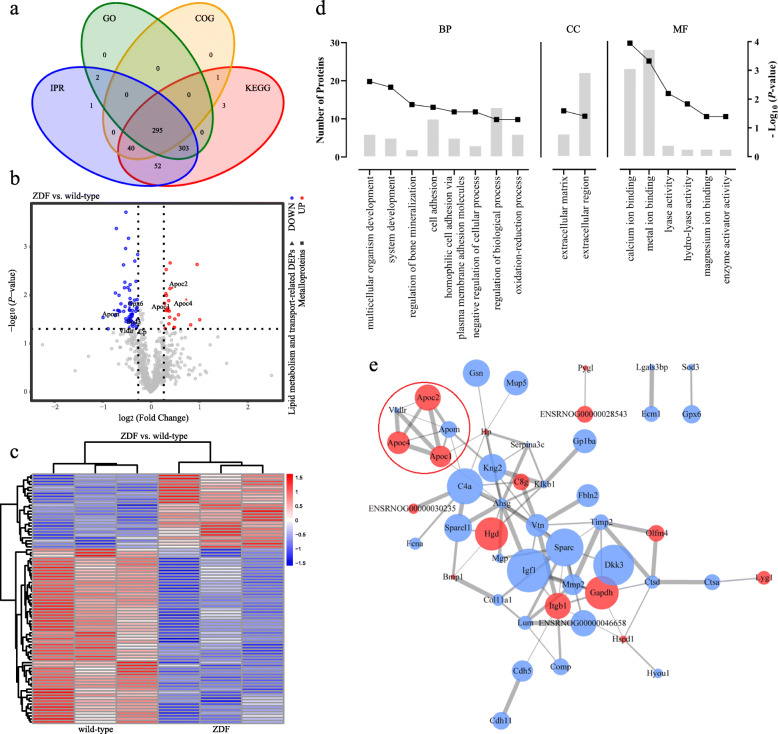
Visualization of differentially expressed proteins (DEP) and enrichment analysis. **a** Function annotations Venn graph of all the identified 697 proteins. **b** Volcano plot of DEPs. Gray in the Volcano plot indicates the proteins with insignificant differences, red indicates up-regulated and blue indicates down-regulated. We use triangles and squares to highlight the lipid metabolism and transport-related DEPs and metalloproteins, respectively. **c** Heatmap of DEPs. Each row is corrected for the Z value. Longitudinal is the clustering of samples and horizontal is the clustering of proteins. The heatmap with annotations are provided in the Additional file 4. **d** Histogram of GO enrichment analyses results. The entries in the histogram are arranged from left to right according to the degree of enrichment, and the curves show the change of enrichment degree. **e** Protein-protein interaction network of DEPs. Each node represents a protein. The up-regulated protein is in red and down-regulated protein is blue. The size of each node is proportional to the -log_10_ *P*-value. The edges represent protein-protein interactions. The width of the edge is proportional to the combined-score in STRING. The module circled by the red line is associated with lipid metabolism and transport

We identified a total of 697 proteins (Fig. 2a). Quantitative data and annotation results of these proteins were detailed in the Additional file 2. Among all the identified proteins, 25 were significantly up-regulated (FC > 1.2 and *P* < 0.05) and 59 were markedly down-regulated (FC < 0.83 and *P* < 0.05) (Fig. 2b). The criteria used in our study was appropriate, which was confirmed by the hierarchical clustering of DEPs (Fig. 2c).

GO function enrichment analysis gave significant enriched GO function entries in the DEPs compared to all identified proteins (Fig. 2d), defining the biological function of the DEPs. GO biological process (BP) analysis found that the DEPs were mainly enriched in multicellular organism development, system development, regulation of bone mineralization, cell adhesion, homophilic cell adhesion via plasma membrane adhesion molecules, negative regulation of cellular process, regulation of biological process, and oxidation-reduction process. In the cell composition (CC) part, the DEPs were involved in the extracellular matrix and extracellular region. In the molecular function (MF) section, the DEPs joined in the calcium ion binding, metal ion binding, lyase activity, hydro-lyase activity, magnesium ion binding, enzyme activator activity.

KEGG pathway enrichment analysis demonstrated that the DEPs were enriched in proteoglycans in cancer, ECM-receptor interaction, HIF-1 signaling pathway, endocrine resistance, RNA degradation, which indicated that those above and T2DM share the same molecular pathways.

### PPI analysis of DEPs raised the need for lipidome

There were 69 common proteins and 89 interactions when we matched the 84 DEPs with proteins in the STRING database (*Rattus norvegicus*). The results were described in detail in the permalink: STRING (https://version-11-0.string-db.org/cgi/network.pl?networkId=fOIDKdXKqgFI. Accessed 28 May 2019). A network containing 15 up-regulated proteins and 33 down-regulated proteins was performed after removing unconnected nodes (Fig. 2e). Four significant modules were constructed by MCODE, one of which was associated with lipid metabolism and transport. Preliminary analysis of these proteins in this module suggested there were some changes in the plasma lipids. So, we conducted plasma lipidomics.

### The screening of DELs

**Fig. 3:**
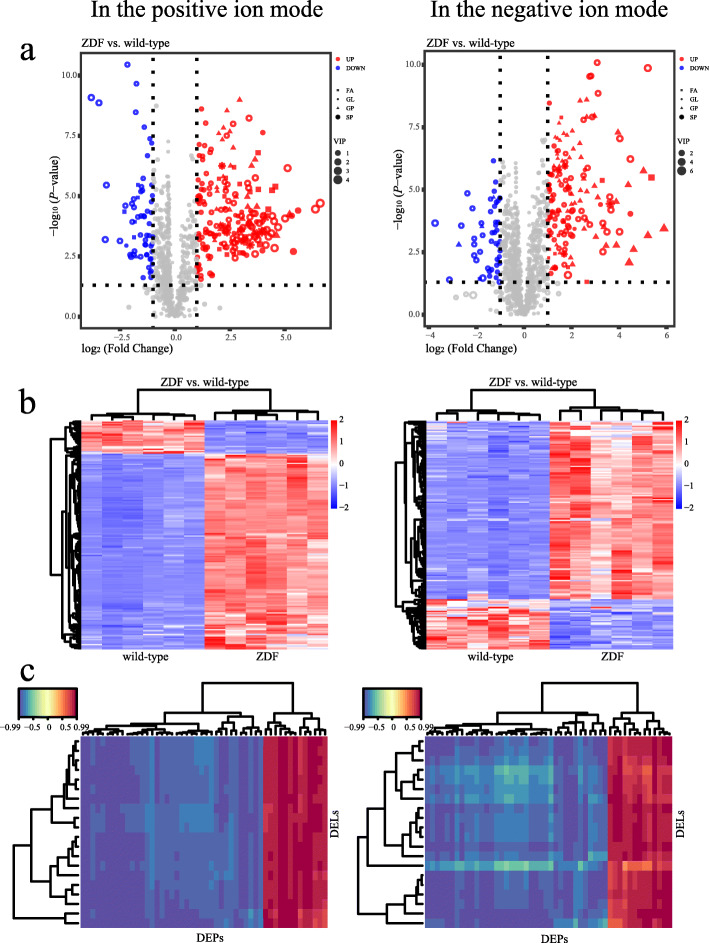
Visualization of differentially expressed lipids (DEL) and their correlation with DEPs. **a** Volcano plot of DELs. Gray in the Volcano plot represents a lipid with no differential expressions, red represents up-regulated and the blue represents down-regulated. We use different shapes to highlight the lipid categories that changed significantly. The size represents the variable importance in the projection (VIP). **b** Heatmap of DELs. Each row is corrected for the Z value. The heatmap with annotations are provided in the Additional file 4. **c** Correlation analysis heatmap of the top 50 DEPs and the top 20 DELs according to the order of FC. The redder the color, the stronger the positive correlation; the bluer the color, the stronger the negative correlation. The heatmap with annotations are provided in the Additional file 4

We identified 1000 lipids in the positive ion mode, of which 153 were substantially up-regulated (VIP > 1.0, FC > 2.0 and *P* < 0.05) and 26 were significantly down-regulated (VIP > 1.0, FC < 0.5 and *P* < 0.05). In the negative ion mode, we identified 1291 lipids, of which 139 were substantially up-regulated and 39 were significantly down-regulated. The quantitative data and statistical analysis results of these lipids were detailed in the Additional file 3. We obtained lipid classification by matching the screened DELs with the Lipidmaps database (http://www.lipidmaps.org), removed unmatched entries and counted the number of DELs accompanied by each classification. The top categories are Glycerolipids (GL), Glycerophospholipids (GP), Fatty Acyls (FA) and Sphingolipids (SP) in the positive ion mode. And in the negative ion mode, they are GP, FA, SP and GL (Fig. 3a). The plasma lipid profile of ZDF rats was different from their basic diet-fed littermate wild-type control (Fig. 3b), and like the plasma protein profile, it could distinguish the state of T2DM.

### Correlation analysis suggested the main reasons for the differences

The expressions of the top 50 DEPs and the top 20 DELs are strongly correlated. A simple statistical display of the absolute value of the Pearson correlation coefficient is as follows: Mean ± SD and Median [IQR], 0.90 ± 0.05 and 0.91 [0.07] in the positive ion mode and 0.86 ± 0.09 and 0.88 [0.11] in the negative ion mode. Correlation analysis showed a high consistency between DEPs and DELs (Fig. 3c). Please refer to the Additional file 4 for the correlation analysis heatmap with detailed DEPs/DELs annotations.

**Fig. 4:**
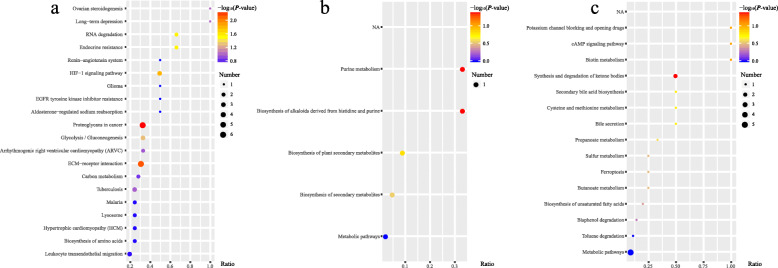
Visualization of KEGG pathway enrichment analysis of DEPs and DELs. **a** Bubble chart of KEGG pathway enrichment analysis of DEPs. Abscissa represents the enrichment degree, the ratio of differences to the number of backgrounds identified in the pathway. The color is proportional to the -log_10_ *P*-value and the size represents the number. **b** Result of DELs in the positive ion mode. **c** Result of DELs in the negative ion mode. (DEPs results show only a part, DELs results are comprehensive)

Based on this, we conducted KEGG pathway enrichment analysis on DELs as did on DEPs aiming to find the main reasons for the differences. The analysis prompted that DELs were enriched in purine metabolism, biosynthesis of alkaloids derived from histidine and purine in the positive ion mode, and in synthesis and degradation of ketone bodies in the negative ion mode. The original *P*-value was then corrected by hypergeometric verification, and the KEGG pathway enrichment results of both DEPs and DELs were compared and reviewed (Fig. 4). We found that metabolism disorder of glycan and lipid plays a significant role in the pathogenesis of T2DM. Besides, the enrichment results of DEPs also suggested oxidative stress and insulin resistance were related to the changes. Table 1 displays the candidate biomarkers related to the mechanism of these differences.

**Table 1:** Candidate biomarkers of T2DM suggested by KEGG pathway enrichment analysis

Pathogenesis (suggested by KEGG pathways)	Candidate biomarkers of T2DM	
	Up-regulated proteins	Down-regulated proteins
Oxidative Stress		ceruloplasmin
		extracellular superoxide dismutase [Cu-Zn]
		glutathione peroxidase 6
Insulin Resistance	glycogen phosphorylase, liver form	insulin-like growth factor 1, isoform CRA_b
	60 kDa heat shock protein, mitochondrial	
Glycan Biosynthesis and Metabolism	glyceraldehyde-3-phosphate dehydrogenase	
	4-trimethylaminobutyraldehyde dehydrogenase	
Lipid Metabolism	apolipoprotein C-I	apolipoprotein M
	apolipoprotein C-II (Predicted)	very low-density lipoprotein receptor
	apolipoprotein C-IV	

## Discussion

Our study here showed that ZDF rats (fa/fa) and their basic diet-fed littermate wild-type rats (fa/+) exhibited different plasma proteins and lipids profiles which could distinguish the diabetic status of rats clearly by the hierarchical clustering of DEPs/DELs. GO function enrichment analysis demonstrated that DEPs were in the extracellular, which gave these proteins the potential to become plasma biomarkers. Furtherly, KEGG pathway enrichment analysis of DEPs and DELs revealed the related mechanisms of T2DM, such as oxidative stress, insulin resistance and metabolic disorders. This was consistent with previous researches [31–33]. Some differentially expressed indicators and their role in KEEG pathways led us to believe that they had the potential to be biomarkers, as follows: Down-regulated ceruloplasmin, extracellular superoxide dismutase [Cu-Zn] and glutathione peroxidase 6 indicated a decrease in antioxidant level [34, 35]. Up-regulated glycogen phosphorylase (liver form), 60 kDa heat shock protein (mitochondrial), and down-regulated insulin-like growth factor 1 (isoform CRA_b) proved a significant insulin resistance [36–38]. Up-regulated glyceraldehyde-3-phosphate dehydrogenase and 4-trimethylaminobutyraldehyde dehydrogenase showed an increasing degree of plasma glycolysis [39]. Up-regulated apolipoprotein C-I and apolipoprotein C-II illustrated blood low-density lipoproteins accumulated in the blood, thereby increasing the risk of cardiovascular complications in diabetes [40, 41].

Importantly, we found two interesting points in these screened biomarkers.

Firstly, three oxidative stress-related markers that we screened, ceruloplasmin, extracellular superoxide dismutase [Cu-Zn] and glutathione peroxidase 6, are all trace elements related-metalloproteins. Ceruloplasmin stores approximately 95% of copper in the blood in a non-diffused state [42] and is linked to iron metabolism [43]. More than half of the patients with aceruloplasminemia (ACP), an autosomal recessive genetic disease caused by mutations in the gene encoding ceruloplasmin, have diabetes as their earliest symptom [44]. And some epidemiological studies use ceruloplasmin to indicate diabetes nephropathy progresses [45–49]. Each subunit of extracellular superoxide dismutase [Cu-Zn] contains a copper ion and a zinc ion, and each of the four subunits of glutathione peroxidase 6 contains a single selenium ion. These metal trace elements play a major part in maintaining the normal function of these proteins [50–52]. So, our study provides evidence for the association of T2DM with trace elements, such as copper, zinc, iron, selenium, through metalloproteins.

The second point is a question of lipid metabolism and transport. A significant module of the DEPs PPI network, which contains three up-regulated proteins, apolipoprotein C-I, apolipoprotein C-II (Predicted) and apolipoprotein C-IV, and two down-regulated proteins, apolipoprotein M and very-low-density lipoprotein receptor, suggests metabolism and transport disorder of lipid. Hierarchical clustering of DELs proves this. Since plasma lipids are greatly influenced by diet, we use the basic diet to feed ZDF rats for 1 week and all animals are fasted for 12 h before collecting plasma samples. And because of this, we don’t screen biomarkers in DELs. It is noteworthy that our results show the association between the apolipoprotein C and T2DM. Since there are limited studies in this area [53, 54], we will pay more attention to the changes in apolipoprotein C during the progress of T2DM in the future.

The pathogenesis of T2DM is complicated. Multi-omics study helps to profoundly understand the molecular mechanisms and explores the possible directions in diagnosis and treatment of it. Screening of plasma biomarkers has unparalleled advantages, as the plasma is more stable and more readily available compared to urine and tissues, respectively [55, 56]. We screened the potential biomarkers of T2DM by comparing the plasma proteins and lipids expression levels in ZDF rats (fa/fa) and their basic diet-fed littermate wild-type controls (fa/+). The comparison method we adopted fully considered the influence of genetics and environments. Although this comparison will overestimate the role of the genetic effects of the leptin receptor gene in T2DM and increase the difficulty of comparison with other similar studies [57, 58], we believe this is a simple and effective comparison strategy when the population’s genetic background is not known clearly. So far, very limited studies have been performed with regard to detection of plasma proteins and lipids profiles in ZDF rats. Therefore, this study may provide a novel strategy to characterize the molecular mechanism of T2DM and search for potential biomarkers [54, 59, 60], despite the fact that this is only at the animal level. It is notable that the samples number is small, although this is sufficient for LC-MS/MS analysis. Increasing samples and verifying the predictability of these candidate biomarkers are the focus of our next work.

## Conclusions

Differentially expressed proteins and lipids in plasma are helpful for early diagnosis and predict the complications of T2DM. Trace elements related-metalloproteins, such as ceruloplasmin, and lipid metabolism and transport-related apolipoprotein C are important in the progression of diabetes and are expected to be candidate plasma biomarkers of T2DM.

## Supplementary information

**Additional file 1 Materials and methods.** Detailed description of materials and methods. **Figure S1.** Quality control of proteomics. **Figure S2.** Quality control of lipidomics. **Figure S3.** Visualization of screening of differentially expressed lipids (DEL).

**Additional file 2 **Differentially expressed proteins (DEP). We considered FC > 1.2 and *P* of FDR validation < 0.05 as DEPs. FC: Fold changes of the mean quantitation (*n* = 3) of the ZDF group to their basic diet-fed littermate wild-type group.

**Additional file 3 **Differentially expressed lipids (DEL). We considered VIP of the PLS-DA model > 1.0, FC > 2.0 and *P* of T-test < 0.05 as DELs. FC: Fold changes of the mean quantitation (*n* = 6) of the ZDF group to their basic diet-fed littermate wild-type group; ROC: Subject operating characteristic curve area; VIP: variable importance in the projection of the first principal component of the PLS-DA model.

**Additional file 4 Figure S4.** Heatmap of DEPs. From the longitudinal clustering, the expression pattern clustering of proteins content between ZDF and their basic diet-fed littermate wild-type control could be seen clearly. **Figure S5.** Heatmap of DELs. The hierarchical clustering of DELs could distinguish ZDF and their basic diet-fed littermate wild-type control. **Figure S6.** Correlation analysis heatmap.

## Data Availability

The datasets used and/or analysed during the current study are available from the corresponding author on reasonable request.

## Abbreviations

T2DM: Type 2 diabetes mellitus
ZDF: Zucker diabetic fatty rats
DEP: Differentially expressed proteins
DEL: Differentially expressed lipids
TMT: Tandem Mass Tags test
LC-MS/MS: Liquid chromatography-tandem mass spectrometry
PSMs: Peptide spectrum matches
FC: Fold change
PPI: Protein–protein interaction
PCA: Principal component analysis
PLS-DA: Partial least squares discriminant analysis
VIP: Variable importance in the projection
ACP: Aceruloplasminemia
QC: Quality control

## References

[1] Cho NH, Shaw JE, Karuranga S, Huang Y, da Rocha Fernandes JD, Ohlrogge AW (2018). IDF diabetes atlas: global estimates of diabetes prevalence for 2017 and projections for 2045. Diabetes Res Clin Pract.

[2] Cole AR, Astell A, Green C, Sutherland C (2007). Molecular connexions between dementia and diabetes. Neurosci Biobehav Rev.

[3] Felber JP, Golay A (2002). Pathways from obesity to diabetes. Int J Obes Relat Metab Disord.

[4] Twig G, Afek A, Derazne E, Tzur D, Cukierman-Yaffe T, Gerstein HC (2014). Diabetes risk among overweight and obese metabolically healthy young adults. Diabetes Care.

[5] Scheen AJ, Van Gaal LF (2014). Combating the dual burden: therapeutic targeting of common pathways in obesity and type 2 diabetes. Lancet Diab Endocrinol.

[6] la Fleur SE, Kalsbeek A. Increased Risk of Diabetes due to Obesity: Does Chronodisruption Play a Role? In: Garaulet M, Ordovás JM, editors.Chronobiology and Obesity. New York: Springer New York; 2013. p. 111-31.

[7] Laaksonen R, Ekroos K, Sysi-Aho M, Hilvo M, Vihervaara T, Kauhanen D (2016). Plasma ceramides predict cardiovascular death in patients with stable coronary artery disease and acute coronary syndromes beyond LDL-cholesterol. Eur Heart J.

[8] Lilamand M, Hourregue C, Paquet C. Interest of biological biomarkers in the diagnostic approach of neurocognitive disorders in the elderly[published online ahead of print, 2020 Mar 10]. Rev Neurol (Paris). 2020;S0035-3787(20)30389-1. 10.1016/j.neurol.2019.12.006. 10.1016/j.neurol.2019.12.006 32169325

[9] Hilvo M, Meikle PJ, Pedersen ER, Tell GS, Dhar I, Brenner H (2020). Development and validation of a ceramide- and phospholipid-based cardiovascular risk estimation score for coronary artery disease patients. Eur Heart J.

[10] Zhang J, Liu J, Qin X (2018). Advances in early biomarkers of diabetic nephropathy. Revista da Associacao Medica Brasileira (1992).

[11] Aghadavod E, Soleimani A, Amirani E, Gholriz Khatami P, Akasheh N, Sharafati Chaleshtori R (2020). Comparison between biomarkers of kidney injury, inflammation, and oxidative stress in patients with diabetic nephropathy and type 2 diabetes mellitus. Iran J Kidney Dis.

[12] Zhang L, Zhang Q (2020). Glycated plasma proteins as more sensitive markers for glycemic control in type 1 diabetes. Proteomics Clin Appl.

[13] Ohneda M, Inman LR, Unger RH (1995). Caloric restriction in obese pre-diabetic rats prevents beta-cell depletion, loss of beta-cell GLUT 2 and glucose incompetence. Diabetologia..

[14] Cefalu WT (2006). Animal models of type 2 diabetes: clinical presentation and pathophysiological relevance to the human condition. ILAR J.

[15] Nugent DA, Smith DM, Jones HB (2008). A review of islet of Langerhans degeneration in rodent models of type 2 diabetes. Toxicol Pathol.

[16] Al-Awar A, Kupai K, Veszelka M, Szűcs G, Attieh Z, Murlasits Z (2016). Experimental Diabetes Mellitus in Different Animal Models. J Diab Res.

[17] Matteucci E, Giampietro O (2008). Proposal open for discussion: defining agreed diagnostic procedures in experimental diabetes research. J Ethnopharmacol.

[18] Ross PL, Huang YN, Marchese JN, Williamson B, Parker K, Hattan S (2004). Multiplexed protein quantitation in Saccharomyces cerevisiae using amine-reactive isobaric tagging reagents. Mol Cell Proteomics.

[19] Wu J, An Y, Pu H, Shan Y, Ren X, An M (2010). Enrichment of serum low-molecular-weight proteins using C18 absorbent under urea/dithiothreitol denatured environment. Anal Biochem.

[20] Wu J, Xie X, Liu Y, He J, Benitez R, Buckanovich RJ (2012). Identification and confirmation of differentially expressed fucosylated glycoproteins in the serum of ovarian cancer patients using a lectin array and LC-MS/MS. J Proteome Res.

[21] Ashburner M, Ball CA, Blake JA, Botstein D, Butler H, Cherry JM (2000). Gene ontology: tool for the unification of biology. The Gene Ontology Consortium. Nat Gene.

[22] Tatusov RL, Fedorova ND, Jackson JD, Jacobs AR, Kiryutin B, Koonin EV (2003). The COG database: an updated version includes eukaryotes. BMC Bioinformatics.

[23] Kanehisa M, Goto S, Kawashima S, Okuno Y, Hattori M (2004). The KEGG resource for deciphering the genome. Nucleic Acids Res.

[24] Kanehisa M, Goto S, Hattori M, Aoki-Kinoshita KF, Itoh M, Kawashima S (2006). From genomics to chemical genomics: new developments in KEGG. Nucleic Acids Res.

[25] Finn RD, Attwood TK, Babbitt PC, Bateman A, Bork P, Bridge AJ (2017). InterPro in 2017-beyond protein family and domain annotations. Nucleic Acids Res.

[26] Boulesteix AL, Strimmer K (2007). Partial least squares: a versatile tool for the analysis of high-dimensional genomic data. Brief Bioinform.

[27] Dumarey M, Smets I, Vander HY (2010). Prediction and interpretation of the antioxidant capacity of green tea from dissimilar chromatographic fingerprints. J Chromatogr B Anal Technol Biomed Life Sci.

[28] Dunn WB, Broadhurst D, Begley P, Zelena E, Francis-McIntyre S, Anderson N (2011). Procedures for large-scale metabolic profiling of serum and plasma using gas chromatography and liquid chromatography coupled to mass spectrometry. Nat Protoc.

[29] Yuan M, Breitkopf SB, Yang X, Asara JM (2012). A positive/negative ion-switching, targeted mass spectrometry-based metabolomics platform for bodily fluids, cells, and fresh and fixed tissue. Nat Protoc.

[30] Wen B, Mei Z, Zeng C, Liu S (2017). metaX: a flexible and comprehensive software for processing metabolomics data. BMC Bioinformatics.

[31] Wada J, Makino H (2013). Inflammation and the pathogenesis of diabetic nephropathy. Clin Sci.

[32] Mohamed J, Nazratun Nafizah AH, Zariyantey AH, Budin SB (2016). Mechanisms of diabetes-induced liver damage: the role of oxidative stress and inflammation. Sultan Qaboos Univ Med J.

[33] Rehman K, Akash MSH (2017). Mechanism of generation of oxidative stress and pathophysiology of type 2 diabetes mellitus: how are they interlinked?. J Cell Biochem.

[34] Yoshimura S, Takekoshi S, Watanabe K, Fujii-Kuriyama Y (1988). Determination of nucleotide sequence of cDNA coding rat glutathione peroxidase and diminished expression of the mRNA in selenium deficient rat liver. Biochem Biophys Res Commun.

[35] Hayes JD, Flanagan JU (2005). Jowsey IRJAB. Glutathione Transferases.

[36] Newgard CB, Nakano K, Hwang PK, Fletterick RJ (1986). Sequence analysis of the cDNA encoding human liver glycogen phosphorylase reveals tissue-specific codon usage. Proc Natl Acad Sci U S A.

[37] Viengchareun S, Le Menuet D, Martinerie L, Munier M, Pascual-Le Tallec L, Lombès M (2007). The mineralocorticoid receptor: insights into its molecular and (patho) physiological biology. Nucl Recept Signal.

[38] Grossmann C, Gekle M (2009). New aspects of rapid aldosterone signaling. Mol Cell Endocrinol.

[39] Sirover MA (2011). On the functional diversity of glyceraldehyde-3-phosphate dehydrogenase: biochemical mechanisms and regulatory control. Biochim Biophys Acta.

[40] Myklebost O, Williamson B, Markham AF, Myklebost SR, Rogers J, Woods DE (1984). The isolation and characterization of cDNA clones for human apolipoprotein CII. J Biol Chem.

[41] Ren G, Kim JY, Smas CM (2012). Identification of RIFL, a novel adipocyte-enriched insulin target gene with a role in lipid metabolism. Am J Physiol Endocrinol Metab.

[42] de Boer IH, Rue TC, Hall YN, Heagerty PJ, Weiss NS, Himmelfarb J (2011). Temporal trends in the prevalence of diabetic kidney disease in the United States. Jama..

[43] Xu X, Pin S, Gathinji M, Fuchs R, Harris ZL (2004). Aceruloplasminemia: an inherited neurodegenerative disease with impairment of iron homeostasis. Ann N Y Acad Sci.

[44] Vroegindeweij LH, van der Beek EH, Boon AJ, Hoogendoorn M, Kievit JA, Wilson JH (2015). Aceruloplasminemia presents as type 1 diabetes in non-obese adults: a detailed case series. Diab Med.

[45] Cunningham J, Leffell M, Mearkle P, Harmatz P (1995). Elevated plasma ceruloplasmin in insulin-dependent diabetes mellitus: evidence for increased oxidative stress as a variable complication. Metab Clin Exp.

[46] Tuominen JA, Ebeling P, Koivisto VA (1998). Long-term lisinopril therapy reduces exercise-induced albuminuria in normoalbuminuric normotensive IDDM patients. Diabetes Care.

[47] Hidaka S, Kaneko O, Shirai M, Kojima K, Igarashi Y, Oda K (1998). Do obesity and non-insulin dependent diabetes mellitus aggravate exercise-induced microproteinuria?. Clinica Chimica Acta.

[48] Quigg RJ (2011). If oxidized LDL immune complexes are relevant in diabetic atherosclerosis, shouldn't they also be relevant in diabetic nephropathy?. Clin Immunol.

[49] Lee MJ, Jung CH, Kang YM, Jang JE, Leem J, Park JY (2015). Serum Ceruloplasmin level as a predictor for the progression of diabetic nephropathy in Korean men with type 2 diabetes mellitus. Diab Metab J.

[50] Fattman CL, Enghild JJ, Crapo JD, Schaefer LM, Valnickova Z, Oury TD (2000). Purification and characterization of extracellular superoxide dismutase in mouse lung. Biochem Biophys Res Commun.

[51] Bowler RP, Nicks M, Tran K, Tanner G, Chang LY, Young SK (2004). Extracellular superoxide dismutase attenuates lipopolysaccharide-induced neutrophilic inflammation. Am J Respir Cell Mol Biol.

[52] Kim H, Morimoto Y, Ogami A, Nagatomo H, Hirohashi M, Oyabu T (2007). Differential expression of EC-SOD, Mn-SOD and CuZn-SOD in rat lung exposed to crystalline silica. J Occup Health.

[53] Adiels M, Taskinen MR, Bjornson E, Andersson L, Matikainen N, Soderlund S (2019). Role of apolipoprotein C-III overproduction in diabetic dyslipidaemia. Diabetes Obes Metab.

[54] Kim SW, Choi JW, Yun JW, Chung IS, Cho HC, Song SE (2019). Proteomics approach to identify serum biomarkers associated with the progression of diabetes in Korean patients with abdominal obesity. PLoS One.

[55] Afkarian M, Bhasin M, Dillon ST, Guerrero MC, Nelson RG, Knowler WC (2010). Optimizing a proteomics platform for urine biomarker discovery. Mol Cell Proteomics.

[56] Greco V, Piras C, Pieroni L, Urbani A (2017). Direct Assessment of Plasma/Serum Sample Quality for Proteomics Biomarker Investigation. Method Mol Biol.

[57] Backman M, Flenkenthaler F, Blutke A, Dahlhoff M, Landstrom E, Renner S (2019). Multi-omics insights into functional alterations of the liver in insulin-deficient diabetes mellitus. Mol Metab.

[58] Zhong M, Wu Y, Ou W, Huang L, Yang L. Identification of key genes involved in type 2 diabetic islet dysfunction: a bioinformatics study. Biosci Rep. 2019;39(5):BSR20182172. 10.1042/BSR20182172 PMC6542763 31088900

[59] Bock C, Coleman M, Collins B, Davis J, Foulds G, Gold L (2004). Photoaptamer arrays applied to multiplexed proteomic analysis. Proteomics..

[60] Chen HM, Lee LC, Hu KY, Tsai WJ, Huang C, Tsay HJ (2018). The application of post-translational modification oriented serum proteomics to assess experimental diabetes with complications. PLoS One.

