# Supplementary material for: Metalloproteins and apolipoprotein C: candidate plasma biomarkers of T2DM screened by comparative proteomics and lipidomics in ZDF rats
Source: Nutr Metab (Lond). 2020 Aug 12;17:66. doi: 10.1186/s12986-020-00488-2 (PMC7425165; doi:10.1186/s12986-020-00488-2)
Supplement: Supplementary file 1 — Additional file 1 Materials and methods. Detailed description of materials and methods. Figure S1. Quality control of proteomics. Figure S2. Quality control of lipidomics. Figure S3. Visualization of screening of differentially expressed lipids (DEL). [file 12986_2020_488_MOESM1_ESM.docx]

**Additional file 1**

**Materials and methods**

**Plasma samples preparation**

The rats were fasted for 12 hours, freed to drink water and activities at the same time. With the implementation of isoflurane inhalation anesthesia, the blood was collected with the EDTAK_2_ negative pressure blood collection tube by abdominal aorta blood. After centrifugation at 3500 rpm, 4°C for 15 min, 1 ml of the upper layer of yellow plasma was transferred to a cryopreservation tube, and 1 ml of DMEM/High glucose buffer was added to shake and mix, and stored in a -80°C refrigerator.

**TMT labeling and LC-MS/MS analysis**

**Total protein extraction.** Samples were individually milled to a powder in a mortar with liquid nitrogen. Mix the powder with lysis buffer containing 50 mM Tris-HCl (pH=8), 8 M Urea and 0.2% SDS. The homogenate was incubated with ultrasonication on ice for 5 min and then centrifuged at 12000 g for 15 min at 4°C. The supernatant was transferred to a clean tube, and protein concentration was determined by a Bradford assay. Added 2 mM DTT and incubated the sample at 56°C for 1 hour following with adding sufficient iodoacetic acid to the sample and incubating for 1 hour protected from light at room temperature. And then added 4 volumes cold acetone to a sample extract, vortexed well, placed sample at -20°C to overnight. Centrifuged and collected pellet to wash twice with cold acetone. Finally dissolved the pellet by dissolution buffer containing 0.1 M triethylammonium bicarbonate (TEAB, pH=8.5) and 8 M urea. Protein concentration was determined by a Bradford assay. Plasma samples were processed via the ProtenMiner^TM^ protein enrichment kit (Bio-Rad) to remove high-abundance proteins.

**Peptides preparation.** The supernatants from each sample, containing precisely 0.1 mg of protein were digested with Trypsin Gold (Promega) at 37°C for 16 hours. After trypsin digestion, peptides were desalted with C18 cartridge to remove the high urea, and desalted peptides were dried by vacuum centrifugation.

**TMT labeling.** Desalted peptides were labeled with TMT6-plex reagents (TMT6plex™ Isobaric Label Reagent Set, Thermo Fisher), following the manufacturer’s instructions. For 0.1 mg of peptide, 1 unit of labeling reagent was used. Peptides were dissolved in 100 μL of 0.1 M TEAB, and the labeling reagent was dissolved in 41 μL of acetonitrile. After incubation for 1 hour, the reaction was stopped with ammonium hydroxide. Differently labeled peptides were mixed equally and then desalted in peptide desalting spin columns (Thermo Fisher, 89852).

**HPLC fractionation.** TMT-labeled peptide mix was fractionated using a C18 column (Waters BEH C18 4.6×250 mm, 5 μm) on a Rigol L3000 HPLC operating at 1 ml/min. The column oven was set as 50°C. Mobile phases A (2% acetonitrile, adjusted pH to 10.0 using ammonium hydroxide) and B (98% acetonitrile, adjusted pH to 10.0 using ammonium hydroxide) were used to develop a gradient elution. The solvent gradient was set as follows: 3% B, 5min; 3-8% B, 0.1 min; 8-18% B, 11.9 min; 18-32% B, 11 min; 32-45% B, 7 min; 45-80% B, 3 min; 80% B, 5 min; 80-5% B, 0.1 min, 5% B, 6.9 min. The tryptic peptides were monitored at UV 214 nm. Eluent was collected every minute and then merged to 15 fractions. The samples were dried under vacuum and reconstituted in 0.1% (v/v) formic acid (FA) in water for subsequent analyses.

**LC-MS/MS analysis.** Shotgun proteomics analyses were performed using an EASY-n LC^TM^ 1200 UHPLC system (Thermo Fisher) coupled to an Orbitrap Q Exactive HF-X mass spectrometer (Thermo Fisher) operating in the data-dependent acquisition (DDA) mode. A sample volume corresponding to 2 μg of total peptides reconstituted in 0.1% FA was injected onto an Acclaim PepMap100 C18 Nano-Trap column (2 cm×100 μm, 5μm). Peptides were separated on a Reprosil-Pur 120 C18-AQ analytical column (15 cm×150 μm, 1.9μm), using a 90 min linear gradient for TMT6-plex from 5 to 100% eluent B (0.1% FA in 80% ACN) in eluent A (0.1% FA in H_2_O) at a flow rate of 600 nL/min. The solvent gradient was set as follows: 5-10% B, 2 min; 10-40% B, 80 min; 40–55% B, 2 min; 55–90% B, 1 min; 90–100% B, 5 min. For DDA, Q-Exactive HF-X mass spectrometer was operated in positive polarity mode with spray voltage of 2.3 kV and capillary temperature of 320°C. Full MS scans from 350 to 1500 m/z were acquired at a resolution of 60000 (at 200 m/z) with an AGC target value of 3×10^6^ and a maximum ion injection time of 20 ms. From the full MS scan, a maximum number of 40 of the most abundant precursor ions were selected for higher energy collisional dissociation (HCD) fragment analysis at a resolution of 15000 for TMT6-plex (at 200 m/z) with an automatic gain control (AGC) target value of 1×10^5^ , a maximum ion injection time of 45 ms, a normalized collision energy of 32%, an intensity threshold of 8.3×10^3^ , and the dynamic exclusion parameter set at 60 s.

**Proteomic database searching.** The spectrometry mass data was directly imported into Proteome Discoverer 2.2 in raw file for database search. The database used was Uniport *Rattus* *Norvegicus* (Accessed 18 January 2019, 36090 sequences). Analysis parameters were as follows: Type of Quantification: Reporter Quantification(TMT 10plex); Enzyme: Trypsin; Instrument: Thermo Q Exactive™ HFX; Max.Missed Cleavage Sites: 2; Precursor Mass Tolerance: 10 ppm; Fragment Mass Tolerance: 0.02 Da; Dynamic Modification: Oxidation/+15.995 Da (M) and TMT 10plex/+229.163 Da (K); N-Terminal Modification: Acetyl/+42.011 Da (N-Terminal) and TMT 10plex/+229.163 Da (N-Terminal); Static Modification: Carbamidomethyl/+57.021 Da (C).

**Lipidomic LC-MS/MS analysis**

**Lipid extraction.** Samples (100 μL) were transferred to a glass tube, and then 900 μL of water, 2 ml of methanol and 0.9 ml of dichloromethane were added, followed by sufficient mixing. After 1.0 ml of water and 0.9 ml of dichloromethane were added, the samples were vortexed to emulsification and centrifuged at 3000 rpm, 4°C for 5 min. The lower dichloromethane layer was subsequently transferred to a fresh glass tube with a glass syringe. The supernatants were added 2 ml of dichloromethane and centrifuged at 3000 rpm, 4°C for 5 min. Merged the collected lower dichloromethane layers and spun in a vacuum concentrator until dry. The dried metabolite pellets were redissolved in 500 μl of methanol/dichloromethane and analyzed by LC-MS/MS. The same amount of each samples was mixed as a quality control (QC) sample and a total of 3 QC samples were used in our study.

**HPLC analysis.** Chromatographic analysis was performed using a Waters ACQUITY UPLC I-Class system. Samples were injected onto a C18 CSH column (100 mm × 2.1 mm, 1.7 μm; Waters) using a 20 min linear gradient at a flow rate of 0.4 ml/min. The column temperature was set at 45 °C. Mobile phase buffer A was acetonitrile/water (1/4) with 10 mM ammonium formate and 0.1% formic acid, whereas buffer B was acetonitrile/isopropanol (1/9) with 10 mM ammonium formate and 0.1% formic acid. The solvent gradient was set as follows: 40% B, initial; 43% B, 2 min; 50% B, 2.1 min; 54% B, 12 min; 70% B, 12.1min; 99% B, 18min; re-equilibration for 2 min at 40% B.

**Mass spectrometry analysis.** After separation by UPLC, mass spectrometry was performed using a Xevo G2-S Q-TOF with an electrospray ionization (ESI) source (Waters, Manchester, UK). Dynamic range enhancement was implemented to the mass spectrometry method of the Xevo G2-S Q-TOF in order to improve isotopic distribution and mass accuracy and reduce high ion intensities. In positive ion-mode, MS parameters were as follows: capillary voltage was set at 2.5 kV, cone voltage at 24 V, source temperature at 100°C, desolvation temperature at 400°C, desolvation gas flow at 800 L/h, and cone gas flow at 50 L/h. Acquisition was performed from m/z 100 to 1500. In negative ion-mode, MS parameters were as follows: capillary voltage was set at 2.5 kV, cone voltage at 25 V, source temperature at 100°C, desolvation temperature at 500°C, desolvation gas flow at 600 L/h, cone gas flow at 10 L/h. Acquisition was performed from m/z 100 to 1500.

**Lipidomic database searching.** The raw data was imported to the Progenesis QI (Waters) to conduct analysis, such as pretreatment, peak alignment, peak integration, peak extraction, multivariate statistical analysis and lipids identification. Lipidmaps (http://www.lipidmaps.org), HMDB (http://www.hmdb.ca), NIST (https://chemdata.nist.gov) and an in-house lipid database were used for metabolites identification. The mass error used was 5 ppm. Peak alignment was performed using the QC sample as a reference group. Mass fraction correction was performed by Lockmass. Data quality control was performed via QC correlation analysis and total samples principal component analysis (PCA).

**Quality control**

The proteomics quality control results after database searching demonstrated that the results had a high reliability (Fig. S1). The 3 QC samples in the lipidomics were correlated closely and the PCA analysis of them can be completely separated from the ZDF and their basic diet-fed wild-type controls (Fig. S2), indicating that the entire processing and analysis procedure were stable. PCA and PLS-DA analysis during differentially expressed lipids screening also verified this conclusion (Fig. S3). In summary, our data is reliable.


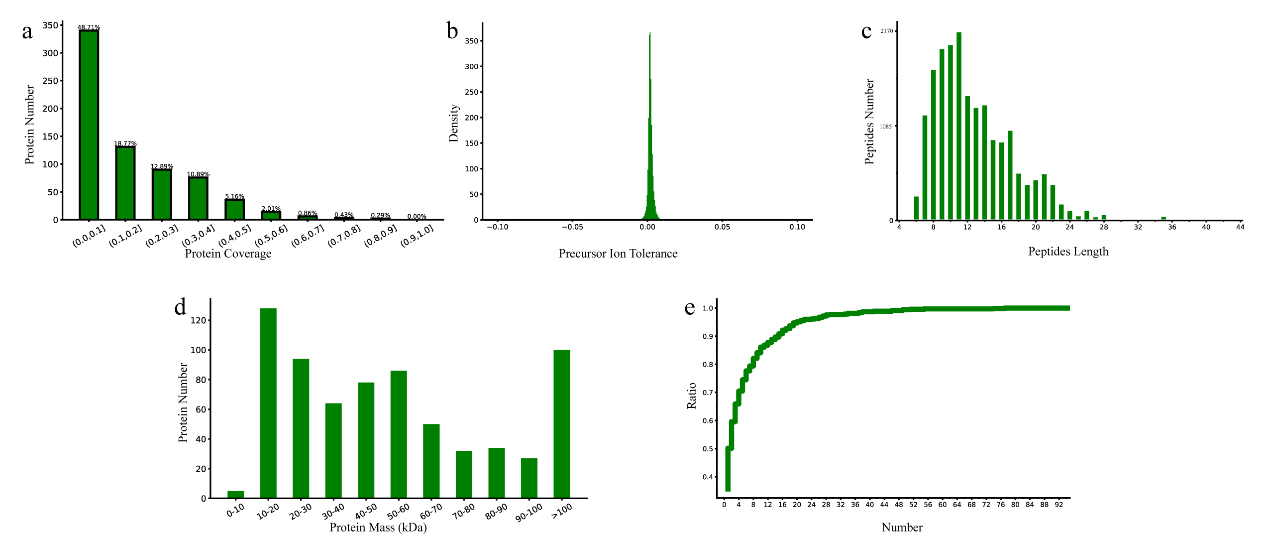


**Fig. S1 Quality control of proteomics.**

**a** Protein coverage distribution. **b** Precursor ion tolerance distribution. **c** Peptides length distribution. **d** Protein mass distribution. **e** Unique peptide number distribution.


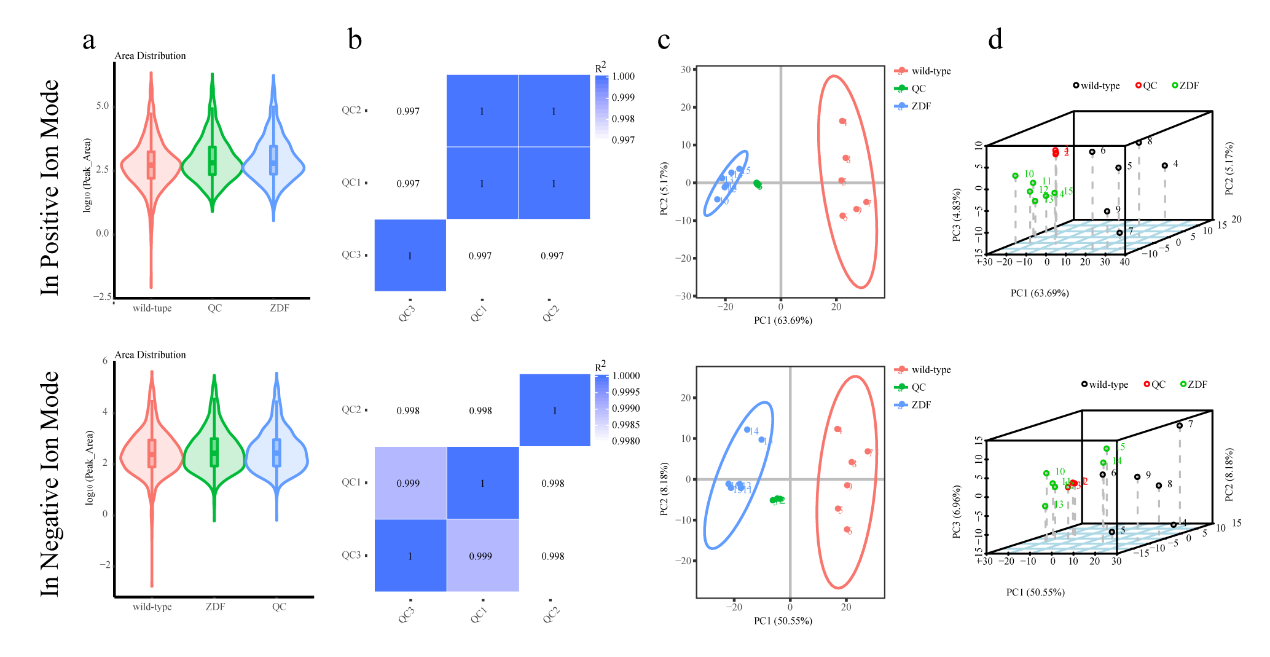


**Fig. S2 Quality control of lipidomics.**

**a** Violin plot of lipids quantitative results. The middle thick line indicates the quartile range. The middle line of it is the median and the thin line extending from it represents the 95% confidence interval. **b** There is a high correlation between the 3 QC samples. R^2^: The square of the Pearson correlation coefficient. **c** Principal component analysis (PCA) of total samples. The abscissa PC1 and the ordinate PC2 represent the scores of the first and second main components, respectively. The scatter color indicates the experimental grouping of the samples. A confidence ellipse of 95% is identified. **d** 3D map of total samples PCA.


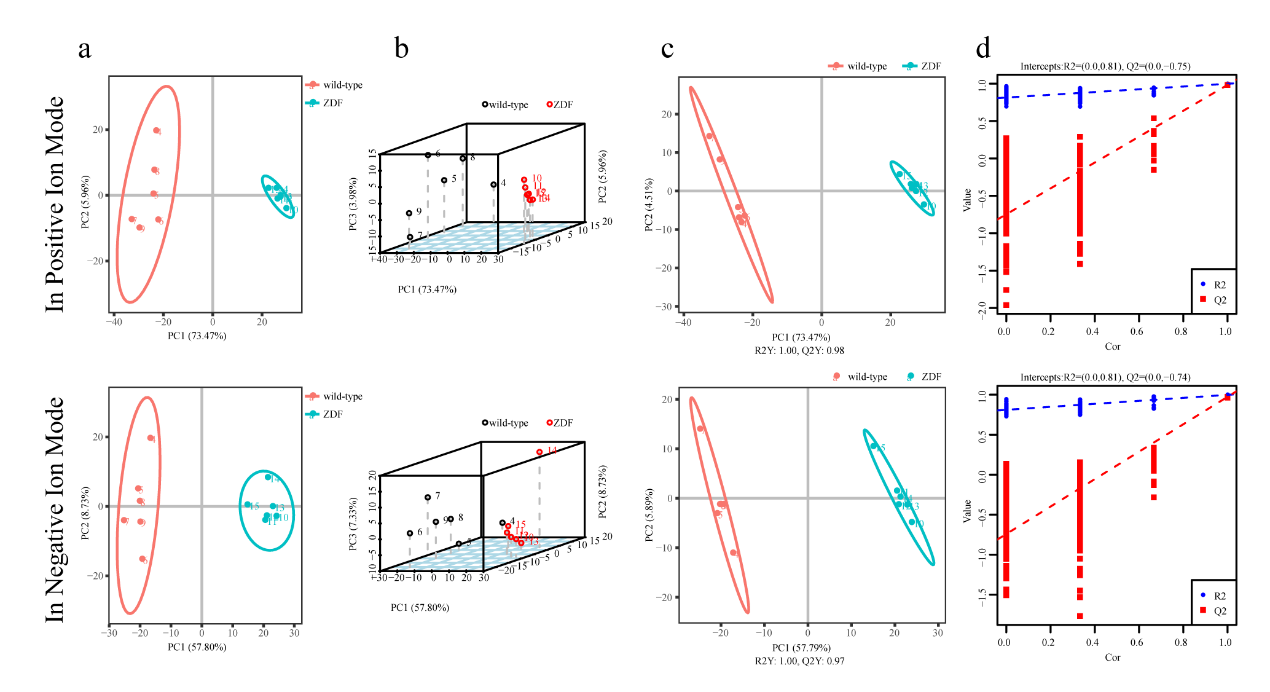


**Fig. S3 Visualization of Differentially Expressed Lipids (DEL) Screening.**

**a** Visualization of principal component analysis (PCA) in 2D map. The abscissa PC1 and the ordinate PC2 represent the scores of the first and second main components, respectively. The scatter color indicates the experimental grouping of the samples. A confidence ellipse of 95% is identified. **b** 3D map of PCA. **c** Score scatter plot of partial least squares discriminant analysis (PLS-DA). The abscissa is the score of the sample on the first principal component. The ordinate is the score of the sample on the second principal component. R2Y represents the interpretation rate of the second principal component of this model. Q2Y represents the prediction rate of this model. **d** Sort check of PLS-DA. The abscissa represents the correlation of the random grouped Y with the original group Y and the ordinate represents the scores of R2 and Q2.
