# Supplementary figures and images for: Metalloproteins and apolipoprotein C: candidate plasma biomarkers of T2DM screened by comparative proteomics and lipidomics in ZDF rats
Source: Nutr Metab (Lond). 2020 Aug 12;17:66. doi: 10.1186/s12986-020-00488-2 (PMC7425165; doi:10.1186/s12986-020-00488-2)

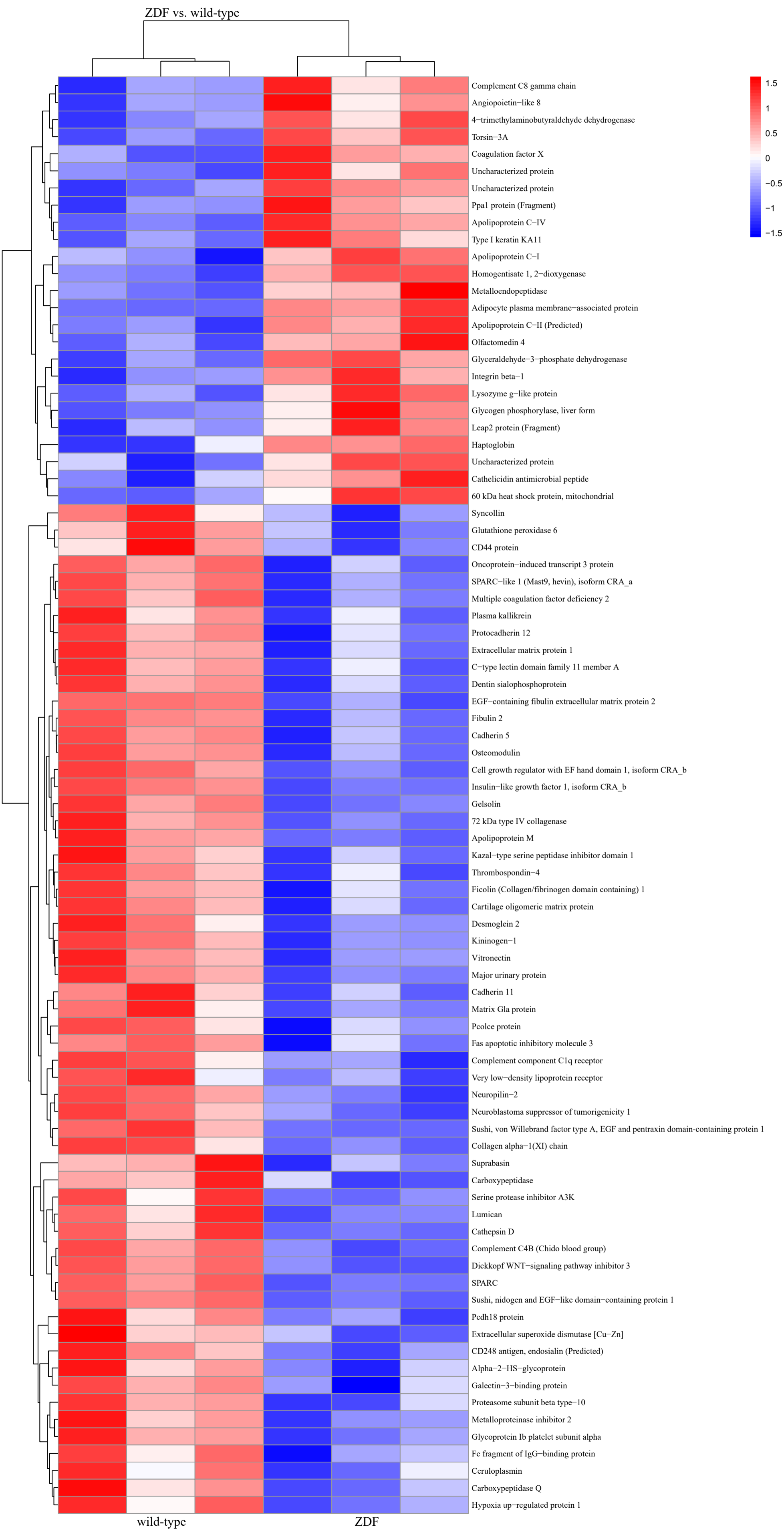

Supplement: Supplementary file 4 — Additional file 4 Figure S4. Heatmap of DEPs. From the longitudinal clustering, the expression pattern clustering of proteins content between ZDF and their basic diet-fed littermate wild-type control could be seen clearly. Figure S5. Heatmap of DELs. The hierarchical clustering of DELs could distinguish ZDF and their basic diet-fed littermate wild-type control. Figure S6. Correlation analysis heatmap. [file 12986_2020_488_MOESM4_ESM.zip › Additional file 4 Fig. S4 Heatmap of DEPs..pdf]

In the negative ion mode

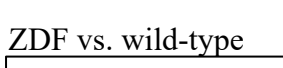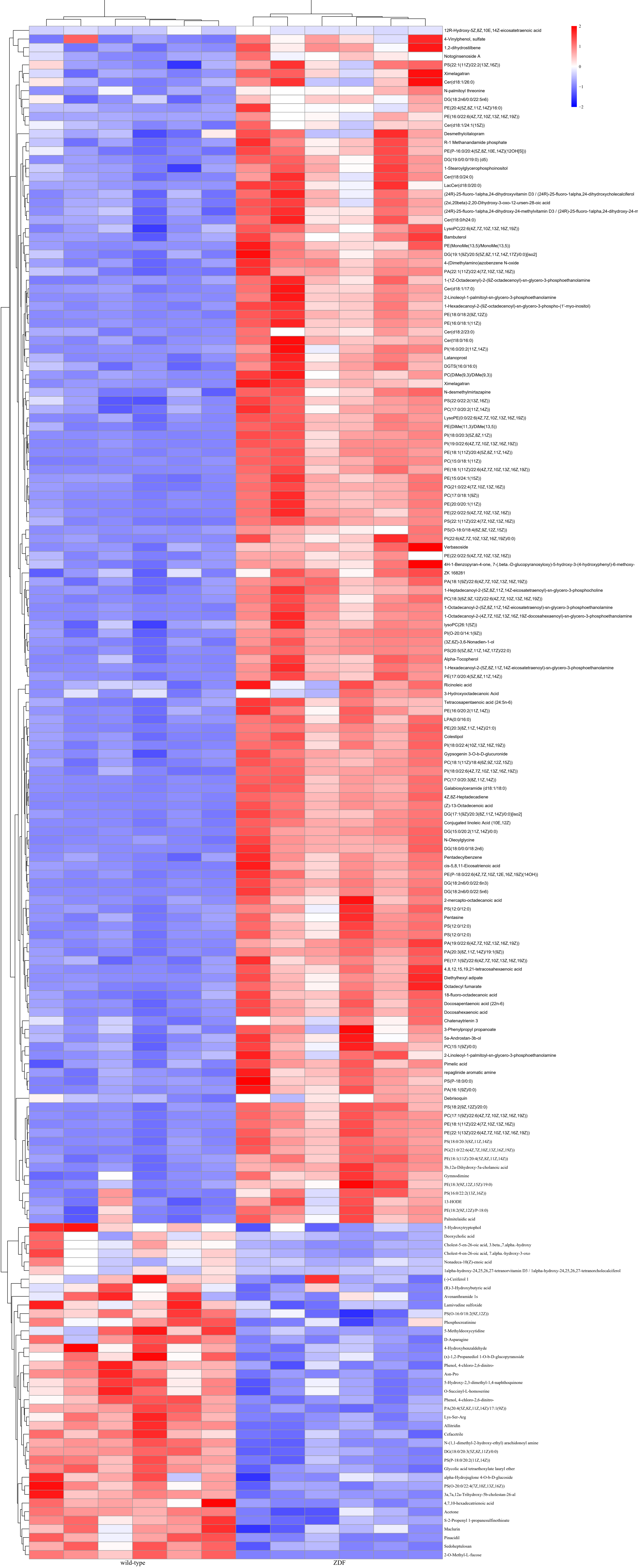

Supplement: Supplementary file 4 — Additional file 4 Figure S4. Heatmap of DEPs. From the longitudinal clustering, the expression pattern clustering of proteins content between ZDF and their basic diet-fed littermate wild-type control could be seen clearly. Figure S5. Heatmap of DELs. The hierarchical clustering of DELs could distinguish ZDF and their basic diet-fed littermate wild-type control. Figure S6. Correlation analysis heatmap. [file 12986_2020_488_MOESM4_ESM.zip › Additional file 4 Fig. S5 Heatmap of DELs..pdf]

In the negative ion mode

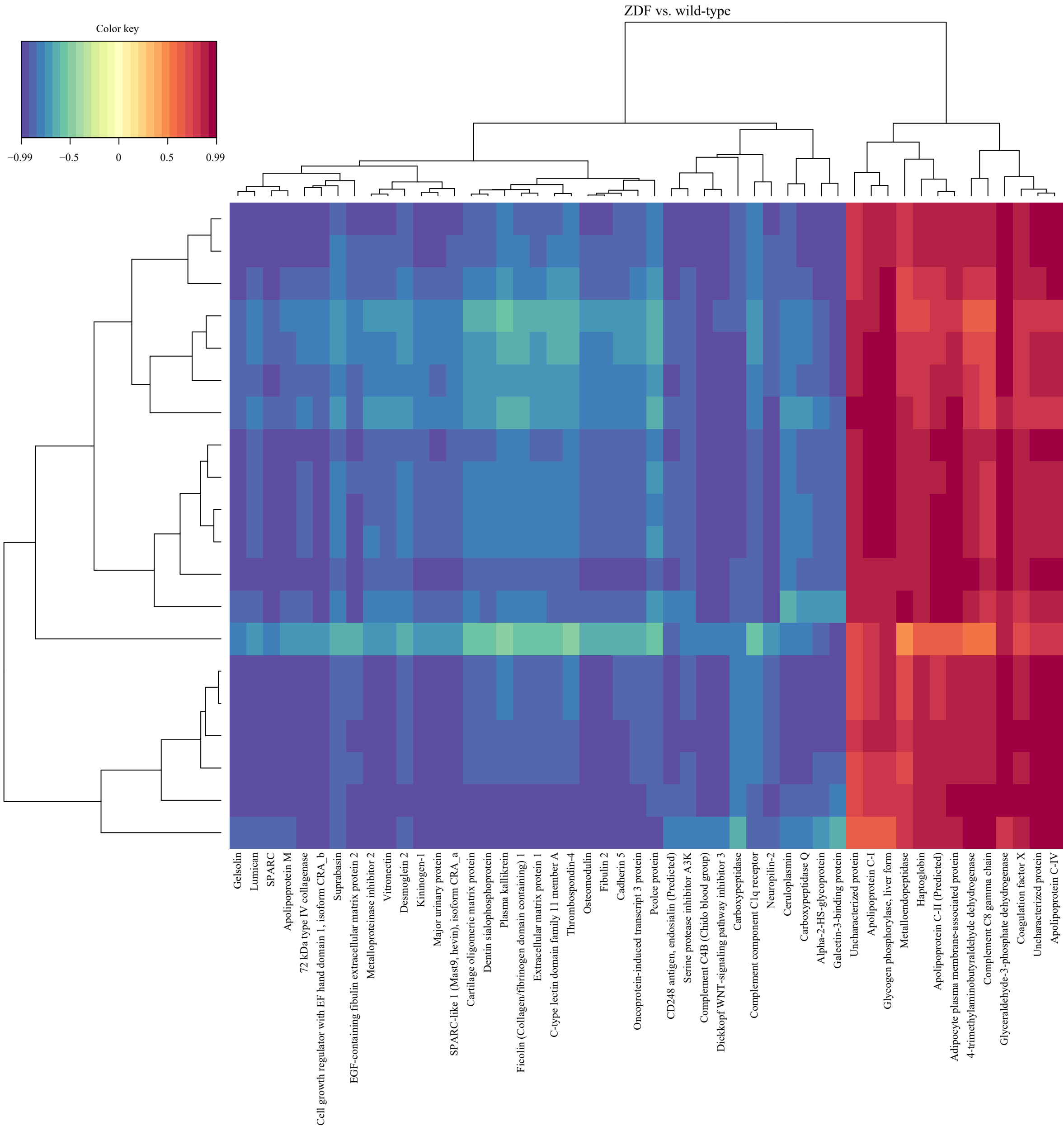

In the positive ion mode

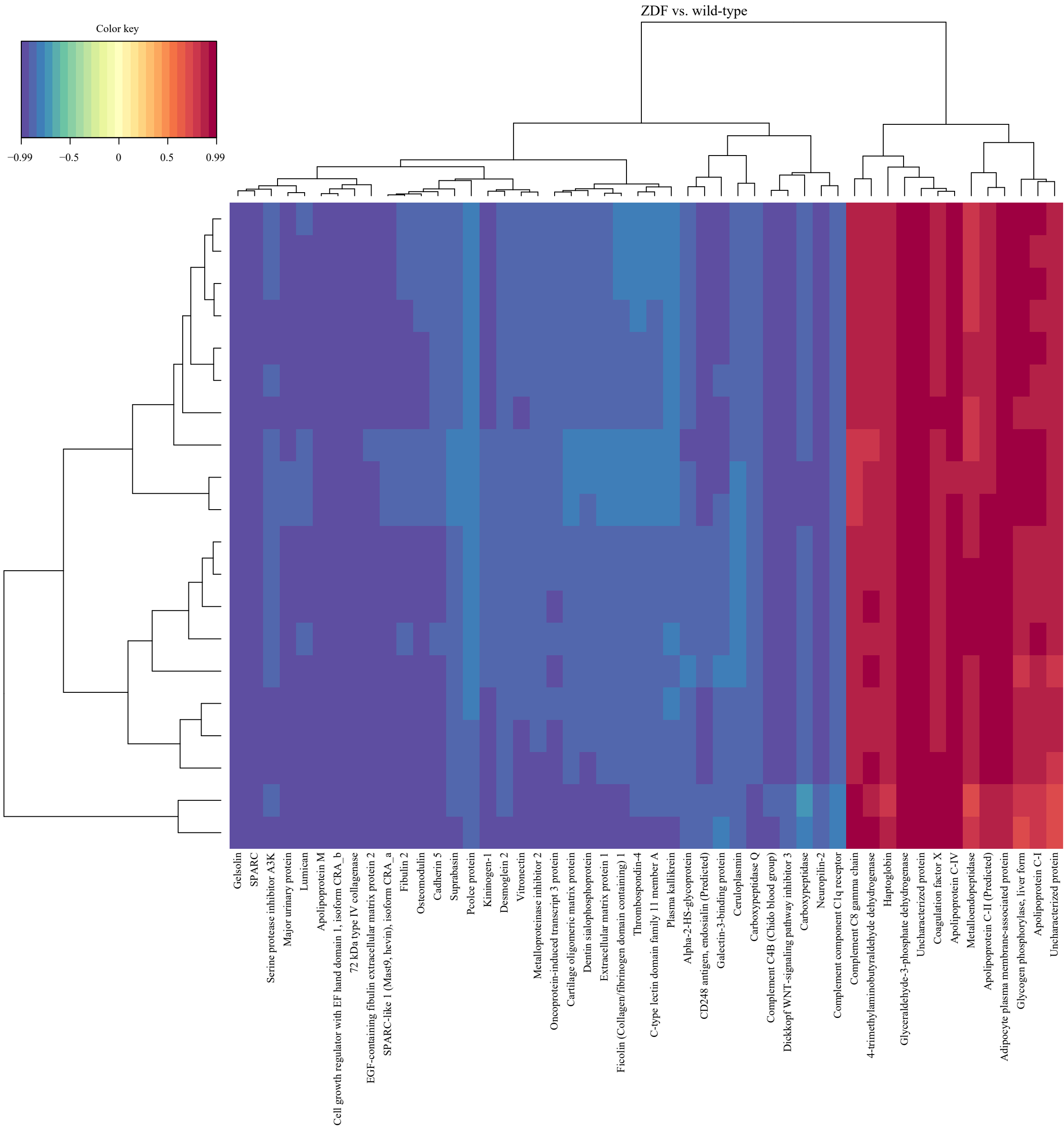

Supplement: Supplementary file 4 — Additional file 4 Figure S4. Heatmap of DEPs. From the longitudinal clustering, the expression pattern clustering of proteins content between ZDF and their basic diet-fed littermate wild-type control could be seen clearly. Figure S5. Heatmap of DELs. The hierarchical clustering of DELs could distinguish ZDF and their basic diet-fed littermate wild-type control. Figure S6. Correlation analysis heatmap. [file 12986_2020_488_MOESM4_ESM.zip › Additional file 4 Fig. S6 Correlation analysis heatmap..pdf]
